# A Systematic Review of Laboratory Evidence for the Abuse Potential of Tramadol in Humans

**DOI:** 10.3389/fpsyt.2019.00704

**Published:** 2019-09-26

**Authors:** Kelly E. Dunn, Cecilia L. Bergeria, Andrew S. Huhn, Eric C. Strain

**Affiliations:** Behavioral Pharmacology Research Unit, Department of Psychiatry and Behavioral Sciences, Johns Hopkins University School of Medicine, Baltimore, MD, United States

**Keywords:** opioid, tramadol, Ultram, abuse, pain

## Abstract

**Background:** Tramadol is an opioid-analgesic that has shown epidemiological evidence of abuse. This review evaluates the evidence for tramadol abuse potential in humans.

**Methods:** A systematic literature search for human abuse liability examinations of tramadol was conducted in September 2018 and yielded 13 total studies. Studies were all within-subject, double-blind, placebo-controlled human laboratory comparisons of tramadol to opioid comparators. Results are organized based upon the route of tramadol administration (oral, parenteral) and the participant population (persons with and without current opioid physical dependence). Outcomes were categorized into self-report ratings of positive and negative effects, observer-ratings of effects, time course of effects, likelihood tramadol was identified as an opioid, and tramadol self-administration.

**Results:** Results indicated the relative abuse potential of tramadol was lower than the opioids to which it was compared. Tramadol produced highest positive effect ratings when administered orally to persons with no opioid physical dependence. Relative to other opioids, it produced substantial negative ratings, generally demonstrated a slower onset of effects, and was less likely to be identified by participants as an opioid, though it did produce a higher rate of self-administration relative to other opioids in the one study reporting that outcome. Results suggest that the abuse potential of tramadol is highest when it is administered orally to non-dependent individuals, and that it likely decreases as the dose increased and when it was administered parentally or to persons with opioid physical dependence.

**Conclusion:** Taken together, individuals may be less likely than with other opioids to escalate tramadol doses, transition from oral to parenteral routes of administration, or continue using tramadol once opioid physical dependence develops. In that way, the human abuse potential of tramadol appears to be different from and lower than other opioid analgesic medications.

## Introduction

### Rationale

Opioid-based analgesic medications are a mainstay for the management of moderate to severe pain throughout the world. Despite the fact that global demand for opioids has increased significantly in the past 20 years (1), the international availability and acceptability of opioids for pain management varies considerably. A recent study reported that the majority of the world’s population has insufficient access to opioids for pain management (2, 3), with only 7.5% of people having adequate opioid access and 66% of people having little to no access (4). Some of the myriad reasons for limited opioid access include differences in provider practices and healthcare system operations, financial barriers, cultural beliefs regarding pain management, and/or variations in opioid-related regulations (5–7). Substantial stigma and opiophobia that further reduce opioid availability and distribution have also been reported throughout African, Latin American, and Eastern European countries (7–11).

The use of opioid analgesic treatment for pain management is complicated by the fact that opioids can produce euphoric effects and are often misused. Human abuse liability studies provide a direct way to evaluate the abuse potential of medications like opioids and provide important data that are used by regional and international agencies to guide regulatory decisions (12). Human abuse liability studies generally collect several important outcome measures, ranging from self-reports of positive and negative drug effects to operant drug self-administration, and compare effects of the medication in question to other prototypical drugs in individuals with and without a history of drug misuse (13–16). International regulatory bodies rely on evidence of drug therapeutic efficacy and abuse potential to determine how medications should be regulated, and the schedule on which a medication is placed can impact its availability for therapeutic use.

### Tramadol

This manuscript reviews human laboratory studies that assessed the abuse potential of tramadol. Tramadol is an analgesic with opioid-like effects (17, 18) that has a unique pharmacokinetic and pharmacodynamic profile relative to other opioids. It is marketed in both immediate and extended-release formulations and is indicated for the treatment of mild to severe pain at doses up to 200 (immediate) or 300 (extended-release) milligrams per day. Tramadol is the only opioid classified as a step 2 medication on the World Health Organization (WHO) analgesic ladder, thereby making it the only opioid-like medication available for the management of moderate and severe pain in countries whose policies limit patient and provider access to step 3 “strong” medications (19, 20).

Tramadol was first synthesized in 1962 and has been commercially available as an analgesic medication since 1977 (21). It is a racemic compound and both enantiomers and their metabolites are physiologically active and contribute to its effect profile (22, 23). Tramadol inhibits serotonin [(+)-*Trans*-T enantiomer] and norepinephrine [(-)- *Trans*-T enantiomer] reuptake (22, 24, 25) before being converted *via* hepatic metabolism to active and inactive metabolites. The M1 metabolite (O-desmethyl-tramadol) binds to the μ-opioid receptor with 300-fold greater affinity than the parent tramadol product (25–27), and is believed to produce the majority of tramadol’s analgesic and euphoric effects (21, 28). Routes of administration that bypass hepatic metabolism produce lower levels of M1, so tramadol that is administered parenterally produces a different pharmacokinetic and pharmacodynamic profile relative to oral administration (29, 30). The contribution of other metabolites (M2-M5) to analgesia and/or opioid-like effects has not been as thoroughly characterized.

Tramadol is similar to other opioids in that continuous exposure can lead to opioid physical dependence and subsequent discontinuation can result in a withdrawal syndrome (31, 32). In contrast to many other opioids, tramadol exerts effects on multiple neurotransmitter systems, including the opioid but also serotonin and norepinephrine systems. The effects of tramadol are only partially blocked by the opioid antagonist naloxone (33, 34), and tramadol displays minimal cross-tolerance with other opioids (32, 35–37), which suggests that its full profile of effects are a combination of its activities on these different systems.

### Epidemiological Trends in Tramadol Abuse

In contrast to most other opioids, tramadol is only regulated at a national (versus international) level, and epidemiological evidence of tramadol abuse appears to vary as a function of its regulatory status. Countries in which a wide variety of opioid products are available generally report low rates of tramadol abuse relative to other opioids (38, 39); however, countries that impose greater restrictions on opioid products and rely more heavily on tramadol for primary pain management often report tramadol abuse. The abuse potential of tramadol has become a particular concern in Egypt and other African countries (40), which have also observed increases in the importation of illicit and adulterated tramadol (41). Tramadol abuse among adolescents and young adults in those countries has been reported to be as high as 8.8% - 12.3%, respectively (42, 43). These countries petitioned the WHO to consider placing tramadol under international control (41) and the WHO Expert Committee on Drug Dependence responded in 2018 by conducting a critical review of tramadol. That review concluded that tramadol should be kept under surveillance but did not warrant international scheduling at that time.

### Objective and Research Question

Subsuming tramadol under international control and/or moving it from a step 2 to step 3 medication to address regional concerns about diversion and misuse could have the consequence of reducing global access to tramadol. This could, in turn, exacerbate the growing international concern that analgesic options for persons in moderate to severe pain are insufficient and inadequate. It is important that empirical examinations of tramadol abuse potential in humans be evaluated to help inform decisions regarding tramadol scheduling. Towards that end, this review updates a previous review on the abuse liability of tramadol that was completed several years ago and which included only one study that evaluated the abuse potential of tramadol administered *via* oral formulation (44).

## Methods

### Search Strategy and Selection Criteria

Automated and manual literature searches for peer-reviewed publications of tramadol human abuse liability studies were conducted. Only studies that experimentally administered tramadol to humans and compared effects to placebo or other opioid comparators were considered for inclusion. A systematic search consistent with the guidelines for systematic reviews outlined by the Preferred Reporting Items for Systematic Reviews and Meta-Analyses (PRISMA) (45) was conducted using PubMed on September 21, 2018. The following search terms were used as key words in the title and abstract, and combined together using the Boolean operators “AND” and “OR”: “tramadol,” “abuse,” “liability,” “potential,” “abuse liability,” “abuse potential,” “dependence,” “self-administration,” “self administration,” “drug discrimination,” “pain AND abuse,” “dopamine,” “reward,” “opioid agonists,” and “opioid antagonists.”

### Inclusion/Exclusion Criteria

Eligible studies were required to have been published in English, have evaluated tramadol abuse potential with an experimental design, and to have been conducted in humans. Studies that reported outcomes related to epidemiological trends, case reports, clinical assessment of tramadol’s therapeutic qualities, guidelines for tramadol prescribing or administration, or retrospective chart reviews; conducted experimental designs with outcomes focused solely on analgesia, adverse effects, neurobiological outcomes (e.g., dopaminergic firing); or reported only genetic or other mediators of tramadol-based effects were excluded. All article abstracts were independently reviewed for eligibility by authors KD, AH, and CB and discrepancies were resolved to consensus.

## Results

### Study Selection and Characteristics

**Figure 1:**
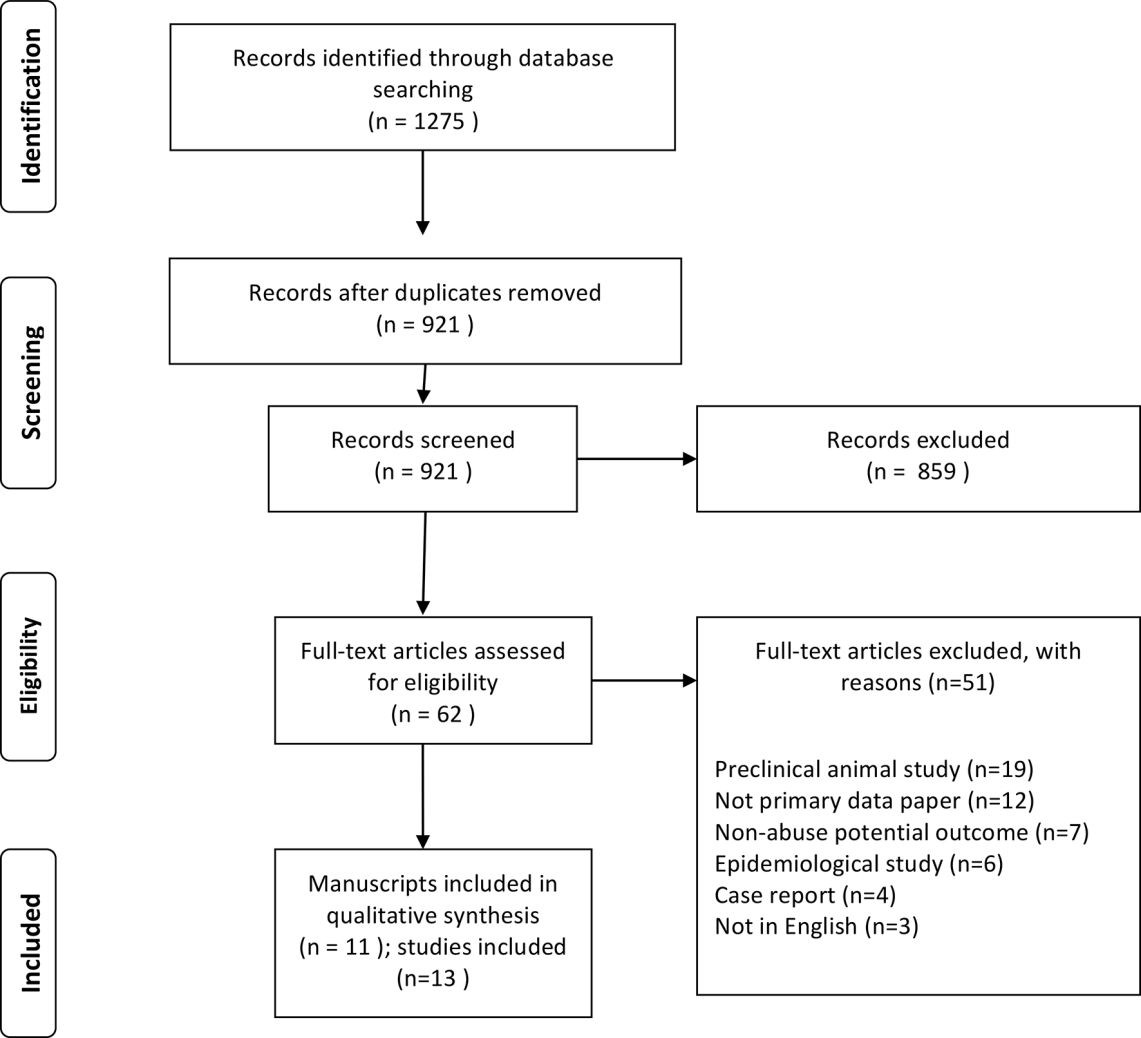
PRISMA Flowchart.

The initial search yielded 921 results; 11 manuscripts met all eligibility criteria and were included in this review (**Figure 1**; **Table 1**). Two manuscripts (31, 44) reported the results of two different studies, resulting in thirteen total studies being reviewed here. The reviewed studies were all within-subject, human laboratory examinations that enrolled small samples, administered study medications in a double-blind manner, and reported several different outcome measures (as recommended for the assessment of opioid abuse liability studies (13)). Most studies reported self-report outcomes, which were collected on visual analog rating (VAS) scales ranging from 0 (not at all) to 100 (highest possible rating) unless otherwise indicated. Some studies also reported whether participants perceived tramadol as an opioid and the time-course of self-report and observer-rated effects. Two studies empirically examined whether double-blind doses of tramadol were perceived by participants to be an opioid using a formal drug discrimination paradigm (46, 47), and one study assessed the degree to which tramadol would be self-administered with an operant task (48). Although studies often evaluated tramadol relative to placebo and other active drug comparators (see **Table 1**), statistical comparisons were generally conducted relative to placebo. As a result, it is only possible to report relative relationships between tramadol and other comparators.

**Table 1:** Summary of Reviewed Studies.

Reference	Physically Dependent on Opioids	Number of Participants	% Male	Tramadol Dose in Milligrams	Tramadol Route of Administration	Comparator Drug and Dose in Milligrams	Monitoring Period
Babalonis et al. (48)	No	9	67%	200, 400	Oral	Placebo Oxycodone (20, 40) Codeine (100, 200)	6 h
Camí et al. (49)	Yes	6	100%	100, 300	Intramuscular	Morphine (60) Placebo	4 h
Carroll et al. (31) (Study 1)	Yes	6	33%	50, 100, 200, 400	Oral	Placebo Hydromorphone (5, 10) Naltrexone (0.6, 1.2)	2 h, 45 min
Carroll et al. (31) (Study 2)	Yes	8	63%	75, 150, 300	Intramuscular	Placebo Hydromorphone (5, 10) Naloxone (0.1, 0.2)	3 h
Das et al. (50)	No	10	100%	100	Intramuscular	Placebo Buprenorphine (0.6)	4 h
Duke et al. (46)	No	8	100%	50, 100, 200, 400	Oral	Hydromorphone (4, 8) Methylphenidate (30, 60)	2.5 h
Epstein et al. (44) (Study 1)	No	10	Not reported	100, 200 (300,700 were initially administered but discontinued for safety)	Intravenous	Placebo Morphine (10, 20)	Not reported
Epstein et al. (44) (Study 2)	No	12	Not reported	175, 350, 700	Oral	Placebo Oxycodone (20, 40)	Not reported
Lofwall et al. (32)	Yes	10	80%	50, 100, 200, 400	Oral	Morphine (7.5, 15; i.m.) Naloxone (0.1, 0.2; i.m.)	4 h
Preston et al. (51)	No	12	100%	75, 150, 300	Intramuscular	Placebo Morphine (15, 30)	12 h
Stoops et al. (52)	No	10	60%	87.5, 175, 350	Oral	Hydromorphone (4, 16)	7 h
Strickland et al. (47)	No	5	100%	25, 50, 100, 150	Oral	Hydromorphone (4) with and without naltrexone (50) pretreatment	5 h
Zacny, (53)	No	12	59%	50, 100	Oral	Morphine (25) Lorazepam (2)	8 h

Comparator drug was delivered by the same route of administration as tramadol unless noted.

The physiological experience of tramadol, and its associated abuse potential, likely varies based on the route by which it is administered (oral vs. parenteral, the only routes examined in these studies) and the population to whom it is administered (non-physically dependent vs. current physical dependence). Since none of the reviewed studies directly compared route or population, outcomes are organized based upon the context of drug exposure. Further, not all studies collected the full array of outcomes discussed below. Given the complexity of the results, the lack of one clear primary outcome that indicates abuse potential, and the need for outcomes to be evaluated in the context of all available evidence, this manuscript summarizes results in the Discussion and **Table 2** (as opposed to the end of each section). **Table 2** categorizes the reviewed studies into the six types of outcomes described in the text, presenting relative results, with the most conservative outcome being endorsed (i.e., any evidence of abuse potential, even at a single dose, is indicated).

**Table 2:** Summary of Abuse Liability of Tramadol Relative to Opioid Comparators.

Abuse liability metrics	Non-physically dependent	Persons with opioid physical dependence
**Positive Effects** Arrow indicates more (↑) or less (↓) positive effects, = designates comparable effects	Oral Administration = Epstein et al. (44) = Duke et al. (46) = Zacny, (53) ↓ Babalonis et al. (48) Parenteral Administration ↓ Epstein et al. (44) ↓ Preston et al. (51)	Oral Administration ↓ Carroll et al. (31) ↓ Lofwall et al. (32) Parenteral Administration ↓ Camí et al. (49) = Carroll et al. (31)
**Negative “Bad” Effects** Arrow indicates more (↑) or less (↓) negative (“bad”) effects, = designates comparable effects	Oral Administration ↑ Stoops et al. (52) ↑ Babalonis et al. (48) = Duke et al. (46) Parenteral Administration ↑ Epstein et al. (44) ↑ Preston et al. (51) = Das et al. (50)	Oral Administration ↑ Lofwall et al. (32) = Carroll et al. (31) Parenteral Administration ↑ Camí et al. (49) ↑ Carroll et al. (31)
**Onset of Effects** Arrow indicates longer (↑) or shorter (↓) time to peak or full duration of effects	Oral Administration ↑ Epstein et al. (44) ↑ Stoops et al. (52) ↑ Babalonis et al. (48) Parenteral Administration ↓ Preston et al. (51) ↓ Das et al. (50)	Oral Administration ↑ Lofwall et al. (32)! Parenteral Administration N/A
**Drug Identification** Arrow indicates greater (↑) or lower (↓) likelihood of identifying tramadol as an opioid	Oral Administration ↓ Stoops et al. (52) ↓ Babalonis et al. (48) Parenteral Administration ↓ Epstein et al. (44)	Oral Administration ↓ Lofwall et al. (32) Parenteral Administration ↓ Camí et al., (49)
**Drug Discrimination** Arrow indicates greater (↑) or lower (↓) likelihood of discriminating tramadol as an opioid, = designates comparable discrimination	Oral Administration = Strickland et al. (47) ↓ Duke et al. (46) Parenteral Administration N/A	Oral Administration N/A Parenteral Administration N/A
**Self-administration** Arrow indicates greater (↑) or lower (↓) likelihood of self-administering tramadol	Oral Administration ↑ Babalonis et al. (48) Parenteral Administration N/A	Oral Administration N/A Parenteral Administration N/A

Direction of results refer to relative relationships between tramadol and the opioids to which it was compared in each study. Epstein et al. (44) and Carroll et al. (31) both reported two different studies that administered tramadol via different routes of administration. Results are simplified here for the purpose of presentation, many small nuances and dose-dependent relationships exist that are not outlined here but are discussed in the manuscript. N/A, non-applicable, no study in that category reported that outcome.

### Synthesized Findings

#### Non-Physically Dependent Participants

Seven studies evaluated tramadol abuse potential in persons without current opioid physical dependence (i.e., non-dependent) who received tramadol *via* oral (*n* = 5) or parenteral (*n* = 2) routes. Despite a lack of physical dependence, participants in the majority of these studies had a history of opioid misuse so were not completely opioid naïve. The exception to this is Zacny, (53), which required a history of recreational drug use but not necessarily opioid use to enroll, resulting in some participants being opioid-naïve.

##### Oral Tramadol

Four studies reported on the positive effect profile of oral tramadol in non-dependent participants. The first study compared tramadol (50, 100 mg) to the opioid morphine (25 mg) and the benzodiazepine lorazepam (2 mg) and reported that both morphine and tramadol (100 mg) increased ratings of flushing, dizziness, and “Feel Drug”; only tramadol (100 mg) was shown to increase ratings of hungry, lightheaded, “Like Drug,” and “Take Again” (53). A second study that compared tramadol (50, 100, 200, 400 mg) to the opioid hydromorphone (4, 8 mg) and the stimulant methylphenidate (30, 60 mg) reported that hydromorphone (8 mg) increased ratings of “High” and “Drug Effect” but that neither tramadol nor methylphenidate increased positive self-report ratings at any dose tested (46). A third study that compared tramadol (175, 350, 700 mg) to the opioid oxycodone (20, 40 mg) found both drugs to increase self-report ratings of “Feel Drug” and “Liking” to the same relative degree (44). Finally, a fourth study observed lower ratings on “Drug Effects” and “Drug Liking” scales following tramadol (200, 400 mg) administration relative to oxycodone (20, 40 mg) and the opioid codeine (100, 200 mg) (48).

Three studies reported on the negative effect profile of oral tramadol in non-dependent participants. In the first study, tramadol (350 mg) produced significantly higher ratings of “Bad Effects,” as well as other potentially aversive outcomes such as “Turning of Stomach,” “Abdominal Pain,” and “Dry Mouth,” relative to hydromorphone (16 mg) (52). More participants also vomited following the high (350 mg) versus lower doses of tramadol (87.5, 175 mg), hydromorphone (4, 16 mg), or placebo (52). The second study reported that a high dose of tramadol (400 mg) and codeine (200 mg) increased ratings of “Bad Effect” significantly more than placebo; lower tramadol (200 mg), codeine (100 mg), or oxycodone (20, 40 mg) doses did not increase “Bad Effect” ratings (48). Finally, the third study found only methylphenidate (60 mg), but not tramadol (50, 100, 200, 400 mg) or hydromorphone (4, 8 mg), increased “Bad Effect” ratings (46).

Two studies reported on the time-course of effects following oral tramadol administration. The results of these studies provided evidence that non-dependent participants may take significantly more time to detect the effects of tramadol relative to oral hydromorphone (52), oral oxycodone (48), and oral codeine (48). In addition, tramadol-induced pupillary constriction, a sign of acute opioid agonist activity, was found to peak 1 h later than hydromorphone (52).

Drug discrimination studies provide some evidence that the interoceptive effects of tramadol are likely dose-dependent and may be different than prototypical opioids. For instance, the two studies that asked participants to guess what type of medication they had received reported that participants did not identify tramadol as an opioid until its dose reached or exceeded 350 mg (48, 52). Two additional studies trained participants to differentiate between a prototypical opioid, placebo, and other medications and then evaluated whether they would categorize tramadol as an opioid when it was administered under double-blind conditions. The first study trained participants to discriminate between oral hydromorphone (8 mg), oral methylphenidate (60 mg), and placebo, and then paid participants to correctly identify several doses of oral tramadol (50, 100, 200, 400 mg), oral hydromorphone (4, 8 mg), oral methylphenidate (30, 60 mg), and placebo (46). Results showed that hydromorphone 4 and 8 mg doses were identified as an opioid 75% and 100% of the time, respectively, whereas tramadol 50, 100, 200, and 400 mg doses were identified as hydromorphone 0, 25%, 63%, and 63% of the time, respectively. The highest dose of tramadol (400 mg) was also likely to be perceived as a stimulant in this study (46). The second study trained participants to discriminate tramadol (100 mg) from placebo and then evaluated their ability to discriminate oral doses of tramadol (25, 50, 100, 150 mg) and hydromorphone (4 mg) following pretreatment with placebo or the opioid antagonist naltrexone (50 mg) (47). Three participants (60% of the sample) were able to successfully discriminate tramadol from placebo in this study, and tramadol was more likely to be identified as an opioid as the dose increased (47).

Only one study has examined tramadol self-administration in humans (48). Participants in this study were provided the opportunity to work for money or 1/7^th^ of a dose of oral tramadol (200, 400 mg), oral oxycodone (20, 40 mg), oral codeine (100, 200 mg), and placebo in a progressive ratio operant task. Results showed that participants were most likely to choose the 400 mg dose of tramadol over money (70% of the dose earned), followed closely by 40 mg of oxycodone (60% of dose earned) (48).

##### Parenteral Tramadol

Studies examining parenteral administration in persons without physical dependence revealed a different profile of tramadol effects relative to oral administration. Two studies reported on the self-report profile of positive effects following parenteral tramadol administration in non-dependent participants. In the first study, morphine [30 mg, intramuscular (i.m.)] increased ratings of “Feel the Drug,” “High,” and “Like the Drug,” whereas tramadol (300 mg, i.m.) increased ratings of “Feel the Drug,” but not “High” or “Like the Drug” (51). Blinded observers in this study also reported that tramadol (150 mg) reduced ratings of “Active” patient behavior more than placebo (51). The second study reported that tramadol [100, 200 mg, intravenous (i.v.)] increased ratings of “Drug Effect” but not “Liking” relative to placebo (44). Both of these studies also reported on the negative effect profile of parenteral tramadol. Participants in the first study reported that tramadol (150, 300 mg) significantly increased their feelings of “Nervousness” and “Stomach Turning,” respectively, but did not increase ratings on a “Dislike the Effect” scale, relative to placebo (51). The second study reported seizures in participants who received intravenous doses of tramadol 300 mg or higher (44).

One study reported on the time-course of parenteral tramadol effects in non-dependent persons. In that study, ratings on a “Feel the Effect” scale were evident within 15 min of both tramadol (i.m.) and morphine (i.m.) administration, but tramadol (i.m.) effects peaked within 1 h, whereas morphine (i.m.) peak effects occurred at 3–4 h. Both drugs continued to produce effects up to 6 h post-dosing (51). Measures of pupillary constriction in that study revealed that effects from tramadol dissipated after 12 h, whereas effects from morphine were still evident at the end of 12-h time observation period (51).

Both of these studies used the Addiction Research Center Inventory (ARCI), a self-report measure that classifies participant responses to drug effects as being representative of different exemplar drug classes, to assess whether participants identified the study drugs as opioids. In both studies, participants categorized morphine but not tramadol as an opioid (44, 51). A third study found that participants categorized both tramadol (i.m.) and the opioid buprenorphine (i.m.) as opioids within 45 min, but that after 240 min only 60% of participants continued to identify tramadol as an opioid versus 100% of participants who received buprenorphine (50).

#### Persons With Opioid Physical Dependence

Four studies reported on outcomes in persons with current opioid physical dependence who received tramadol *via* oral (*n* = 2) or parenteral (*n* = 2) routes.

##### Oral Tramadol

Two studies reported on the positive subjective effect profile of oral tramadol in participants with opioid physical dependence. The first study did not observe an effect of oral tramadol (50, 100, 200, 400 mg) or hydromorphone (5, 10 mg) on self-reported or blinded observer ratings relative to placebo (31). The second study reported that oral tramadol (200, 400 mg) produced significantly higher ratings of “Any Drug Effects” than placebo but did not increase ratings of “High,” “Good Effects,” or “Like the Drug” (32).

The only study that examined the bad effect profile of oral tramadol administration in persons with opioid physical dependence reported that tramadol (200, 400 mg) increased ratings on “Bad Effects” and “Sick” scales significantly more than placebo and at levels that approximated those observed following administration of the opioid antagonist naloxone (0.1 mg, i.m.) (32).

Only one study described the time-course of effects following oral tramadol administration to persons with opioid physical dependence. That study compared oral tramadol (50, 100, 200, 400 mg) to morphine (7.5, 15 mg, i.m.) and naloxone (0.1, 0.2 mg, i.m.) (32). The onset of tramadol’s peak effects were observed to emerge later (45–60 min) than those for morphine (30 min) and naloxone (30 min). Peak effects also ended earlier for tramadol than for the morphine and naloxone comparators. Finally, ratings on a “Bad Effect” scale were observed to begin earlier than were ratings on a “Good Effect” scale (32).

The only study to report drug identification outcomes for oral tramadol among persons with opioid physical dependence reported that participants readily categorized morphine (7.5, 15 mg; i.m.) as an opioid but that participants did not reliably categorize oral tramadol doses (50, 100, 200, 400 mg) as opioid agonists; rather, they were categorized as a myriad of different medications (e.g., antidepressants, opioid antagonists, and placebo) (32).

##### Parenteral Tramadol Administration

Two studies have compared effects of tramadol administered *via* parenteral routes to persons with opioid physical dependence. The first study reported that morphine (dose not reported) but not tramadol (300 mg, i.m.) led to increased ratings on a “Good Effect” scale in persons being maintained on the opioid methadone (49). Interestingly, participants in that study rated “Any Effect” and “Liking” scales the highest following administration of 300 mg of tramadol (i.m.), and rated “High” and “Good Effect” scales the highest following administration of 100 mg tramadol (i.m.), suggesting the relationship between tramadol dose and positive effects in this study was not linear (49). A second study that compared tramadol (75, 150, 300 mg, i.m.) to hydromorphone (5, 10 mg, i.m.) observed no differences between tramadol and hydromorphone from placebo on any of the drug effect or opioid agonist ratings scales (31).

Two studies examining parenteral administration of tramadol to persons with opioid physical dependence reported on the profile of negative drug effects. In the first study, tramadol (300 mg, i.m.) significantly increased ratings on a “Bad Effect” rating scale relative to placebo (49), and the second study observed increases on a “Bad Effect” scale following tramadol (150 mg, i.m.) but not hydromorphone (5, 10 mg, i.m.) (31). The latter study also reported that the ratings on the “Bad Effect” scale produced by tramadol were equivalent to the “Bad Effect” ratings produced by a 0.6 mg dose of oral naltrexone (31).

Finally, the only drug identification study conducted with parenteral tramadol in persons with opioid physical dependence reported that participants did not categorize tramadol (100, 300 mg, i.m.) as an opioid significantly more than placebo on the Addiction Research Center Inventory (49).

## Summary of Main Findings

This review summarized 13 human laboratory studies that empirically examined the abuse potential of tramadol. The studies reviewed here followed recommended assessment guidelines by comparing tramadol to other prototypical μ-opioid receptor agonists and antagonists in controlled experimental settings with persons who did and did not have current opioid physical dependence (13–16). Overall, the reviewed data provide evidence that tramadol has a risk for abuse, but that its risk is generally lower than most of the opioids to which it was compared. The abuse potential of tramadol appears to vary based upon the route by which it is administered and whether the population being studied is physically dependent on opioids.

Human abuse liability studies examine the relative likelihood that a medication will be abused by comparing it to a known standard on a comprehensive array of domains that are known to impact escalation from drug use to misuse and abuse. For instance, drugs that produce a greater magnitude and faster onset of positive effects, as well as a lower number of negative effects, have been identified as having greater abuse risk (54, 55). The risk of abuse also increases over time, when individuals begin consuming larger doses and/or transition from oral to injected routes of administration in order to surmount increases in their level of opioid tolerance. As this happens, impulsive opioid use is believed to gradually transition to compulsive use and opioid use disorder (56). The data reviewed here suggest that many of the effects produced by tramadol are different than those produced by other opioids. The fact that tramadol produces more bad effects as the dose increases and has fewer good effects when it is administered *via* injection, relative to other opioids, suggests that persons may be less willing to escalate tramadol use relative to these comparators, though this remains an empirical question. These features are summarized in detail below.

First, the majority of studies reported that ratings of positive effects following tramadol administration were equivalent or lower than the opioids to which it was compared and, in contrast to other drugs, ratings of positive effects were highest when tramadol was administered orally to individuals who were not physically dependent on opioids. This is likely due to the fact that oral administration results in maximal conversion of the M1 metabolite that confers potent opioid effects. With the exception of one study conducted among opioid-naïve persons that found a 100 mg dose of oral tramadol increased positive ratings relative to placebo (53), the majority of studies evaluating oral tramadol did not observe positive effects in persons without opioid physical dependence until the doses became large (e.g., = / > 350 mg). The fact that tramadol did increase ratings on “Drug Effect” but not “Drug Liking” scales in some studies indicates participants could detect its effects but still rated them as being of lower magnitude than those produced by opioid comparators. Tramadol also appeared to produce non-linear dose effects in some of the studies reviewed. This was particularly evident in a study of individuals with opioid physical dependence who received i.m. tramadol that reported a 100 mg dose of tramadol produced higher ratings on “High” and “Good Effect” scales than a 300 mg dose, though the 300 mg dose did increase ratings on “Liking” and “Bad Effects” scales in that study (49). This non-linear pattern was not observed following administration of the comparator opioids, wherein participant ratings increased in a dose-dependent manner that is hypothesized to contribute to dose escalation and transition from impulsive to compulsive use. Interestingly, the drug identification data suggested that the population who provided the highest ratings for tramadol on “Liking” scales (i.e., non-dependent participants receiving oral tramadol) generally only identified tramadol as an opioid at high doses. When lower doses of tramadol were administered *via* non-oral routes or to persons with opioid physical dependence, participants tended to rate it as being similar to placebo or other non-opioid compounds (e.g., stimulants, antidepressants).

Another characteristic that distinguished tramadol from other opioids in these studies is that it produced a prominent negative (or “Bad Effect”) profile that either preceded (32) or emerged concurrent with its positive effect profile. With the exception of oral codeine, this outcome was not observed for the other opioid comparators. Of particular importance is that tramadol produced negative effects in persons with and without opioid physical dependence and following both oral and parenteral routes of administration. This is in contrast to its positive effect profile, which was prominently observed in persons without physical dependence who received oral tramadol. Further, studies that directly compared tramadol (oral) to the opioid antagonists naloxone (32) or naltrexone (31) found that ratings on “Bad Effect” scales following tramadol administration were similar to low levels of these drugs. In addition, one study reported a higher incidence of vomiting in response to oral tramadol versus oral hydromorphone or placebo (52), and administration of i.v. tramadol at doses ≥300 mg produced seizures (44). These results are consistent with extant evidence that high doses of tramadol can produce nausea/vomiting, seizures, CNS depression, and unconsciousness (57, 58). The fact that tramadol increases self-reports of negative effects, independent of the route by which it was administered or the opioid tolerance level of the participant, differentiates it from most of the opioids evaluated here and would likely discourage abuse-related dosing escalation of tramadol, thus limiting its abuse potential.

Tramadol also displayed a delayed time course of effects relative to other opioids. With the exception of one study of non-dependent participants who received i.m. tramadol (51), every study that reported on tramadol’s time course of effects stated that positive effects emerged later for tramadol than for comparator opioids. Evidence suggests that drugs with a faster onset of effects are often perceived as more reinforcing, thereby increasing their associated potential for abuse (54, 55). The fact that most of the studies reviewed here observed positive effects to emerge more slowly following tramadol administration as compared to other opioids suggests that tramadol abuse potential may be lower than the opioid comparators.

### Limitations

There are some limitations in the conclusions that can be reached from the studies reviewed here. First, a limited number of studies were conducted on this topic and the studies reviewed did not uniformly report on the same outcomes, resulting in some outcomes having limited data available to inform the assessment of abuse potential. Although this is consistent with abuse liability studies, which generally lack a single primary outcome and aim to assess a range of outcomes to examine all available evidence, this approach makes it difficult to compare across studies. Second, the only study that assessed drug self-administration (48) found that participants were more likely to self-administer oral tramadol (400 mg) than all comparator opioids, which would suggest it has higher abuse potential than those opioids. Yet these results conflicted with additional data from that same study that reported lower peak positive effects, higher “Bad Effect” ratings, and a slower onset of positive effects following tramadol than the opioid comparators, all of which would otherwise suggest a reduced potential for abuse. Notable discrepancies between self-administration and other assays within the same study have been observed for other drugs (59). The fact that only one study examined tramadol self-administration makes it difficult to determine how to interpret these conflicting results. The reviewed studies also monitored the effects of tramadol for variable lengths of time. The only study that collected ratings for as long as 12 h reported it took that long for effects from parenteral (i.m.) administration of tramadol to return to baseline levels, and other studies that evaluated oral tramadol effects for 6 (48) or 7 (52) h did not see effects return to baseline before the end of the monitoring period. This brings into question whether the full array of effects from tramadol or the M1 metabolite were appropriately characterized in these studies, given the evidence that oral tramadol might confer the greatest abuse potential.

In addition, all but one study was conducted in persons who had an active or past history of opioid misuse, suggesting the majority of outcomes described here resulted from persons who had an established history of subjectively detecting positive opioid effects. The exception to this is Zacny, (53), which enrolled persons with a history of recreational drug use but did not require previous opioid exposure, though participants in that study may still have had an increased profile of abuse risk relative to persons with no previous history of recreational drug use. The degree to which these results would generalize to individuals who had not previously misused a drug or who were receiving tramadol for pain management is therefore not clear. Several characteristics that may impact abuse potential were also not fully examined by these studies. For instance, none of the studies evaluated outcomes based upon differences in cytochrome (CYP) 2D6 enzyme status. CYP2D6 is responsible for M1 conversion and is highly polymorphic, and individuals genetically classified as a poor, intermediate, extensive, or ultra-rapid CYP2D6 metabolizers might experience different pharmacokinetic, pharmacodynamic, analgesic, and side-effect profiles related to interactions between the M1 metabolite and the μ-opioid receptor (29, 60–66). In addition, none of the studies directly compared oral and parenteral tramadol, or the experience of persons who did and did not have opioid physical dependence, or differences in effects between men and women (see **Table 1**). Moreover, though consistent with within-subject, human laboratory studies, statistical comparisons were generally made between active drug and placebo rather than between active drug conditions, and studies reported numerous outcomes related to abuse potential rather than powering the study for a single primary outcome, which limits the types of conclusions that can be drawn from these data. Finally, despite best efforts, the results of laboratory-based examinations of abuse potential do not always correspond to misuse in real-world settings, which is heavily impacted by the availability of the drug and potential alternatives, cost of a drug, and other cultural/societal factors that are not generally captured in laboratory settings (67).

### Conclusions

All medications confer a risk-benefit ratio that patients, providers, and regulatory agencies must consider when deciding upon appropriate treatment strategies. The treatment approach for acute and chronic pain is especially nuanced because opioid analgesics are an important and crucial aspect of pain management that have an inherent risk of misuse and abuse, and associated morbidity, mortality, and societal consequences. Tramadol is an analgesic that acts on multiple transmitter systems and is a mainstay for the management of moderate to severe pain throughout the world. Recent escalations in regional rates of tramadol abuse have prompted questions about its regulatory status. Thirteen human laboratory studies that rigorously evaluated the abuse potential of tramadol relative to other opioid receptor agonists were reviewed here to support informed discussions about tramadol regulation. These data suggest that tramadol confers an equal or lower risk of abuse than the opioids to which it was compared, that its greatest risk for abuse is when it is being administered *via* oral formulations to non-dependent individuals, and that the likelihood it will be abused appears to decrease as the dose increases or when it is administered parentally. These latter characteristics differentiate tramadol from comparator opioids. The effects of tramadol were also reduced in persons with current opioid physical dependence, relative to persons without dependence, and independent of the route by which it was administered. This suggests that tramadol is less likely than the opioids to which it was compared to be abused in persons who are using other opioids (either through licit or illicit means). Together, these data suggest that individuals may be less likely to escalate tramadol doses or routes of administration relative to other opioids, or to continue using tramadol once opioid physical dependence develops. The present literature therefore suggests that the human abuse potential of tramadol is different from and lower than other opioid agonists.

## Author Contributions

KD, CB, and AH conducted the systematic search and identified articles. All authors contributed to the draft synthesis and summary. All authors have contributed to this final draft and support its submission.

## Funding

Salary support for the investigators on this review was provided by the following grants from the National Institute on Drug Abuse: R01DA042751, R01DA035246, R01DA040644, R34DA042926, T32DA007209.

## Conflict of Interest

In the past 3 years, KD has served as a consultant for Beckley Canopy Therapeutics and Grünenthal. Grünenthal manufactures tramadol but did not contribute to this review. AH has received salary support from Ashley Addiction Treatment through Johns Hopkins University. ES has served as a consultant or served on advisory boards for Indivior, The Oak Group, Caron, Innocoll, Otsuka Pharmaceutical, Analgesic Solutions, and Pinney Associates, and has received research funding through Johns Hopkins University from Alkermes.

The remaining author declares that the research was conducted in the absence of any commercial or financial relationships that could be construed as a potential conflict of interest.
